# Chemical and Enzymatic Approaches to Carbohydrate-Derived Spiroketals: Di-d-Fructose Dianhydrides (DFAs)

**DOI:** 10.3390/molecules13081640

**Published:** 2008-08-12

**Authors:** M. Isabel García-Moreno, Juan M. Benito, Carmen Ortiz Mellet, José M. García Fernández

**Affiliations:** 1 Departamento de Química Orgánica, Facultad de Química, Universidad de Sevilla, Profesor García González 1, 41012 Sevilla, Spain; E-mail: isagar@us.es; 2 Instituto de Investigaciones Químicas, CSIC – Universidad de Sevilla, Américo Vespucio 49, Isla de la Cartuja, 41092 Sevilla, Spain; E-mail: juanmab@iiq.csic.es

**Keywords:** Spirocompounds, stereoselective synthesis, difructose dianhydrides, fructose, inulin, levan

## Abstract

Di-d-fructose dianhydrides (DFAs) comprise a unique family of stereoisomeric spiro-tricyclic disaccharides formed upon thermal and/or acidic activation of sucrose- and/or d-fructose-rich materials. The recent discovery of the presence of DFAs in food products and their remarkable nutritional features has attracted considerable interest from the food industry. DFAs behave as low-caloric sweeteners and have proven to exert beneficial prebiotic nutritional functions, favouring the growth of *Bifidobacterium* spp. In the era of functional foods, investigation of the beneficial properties of DFAs has become an important issue. However, the complexity of the DFA mixtures formed during caramelization or roasting of carbohydrates by traditional procedures (up to 14 diastereomeric spiroketal cores) makes evaluation of their individual properties a difficult challenge. Great effort has gone into the development of efficient procedures to obtain DFAs in pure form at laboratory and industrial scale. This paper is devoted to review the recent advances in the stereoselective synthesis of DFAs by means of chemical and enzymatic approaches, their scope, limitations, and complementarities.

## 1. Introduction

Di-d-fructose dianhydrides (DFAs) comprise a diverse family of mono- or dispirocyclic acetals obtained by condensation of two fructose molecules, with formation of two reciprocal glycosidic linkages. Their basic mono- or bis-spiroketal framework resembles that found in many relevant natural products, including steroidal saponins, polyether ionophores, macrolide antibiotics, insect pheromones, and toxic metabolites from algae and fungi [1,2,3,4,5], and is the target of much synthetic effort [6,7,8,9,10,11,12,13,14]. DFAs are formed upon thermal and/or acidic activation of sucrose- and/or d-fructose-rich materials. The first evidence of DFA formation dates back to the XIX^th^ century, with the observation that the hydrolysate of inulin with dilute acid shows reduced levorotation as compared with pure fructose [15]. In the late 1920s some DFAs were isolated and, to some extent, characterised as dextrorotatory, unfermentable and non-reducing sugars [16,17]. The existence of a battery of DFA diastereomers differing in the anomeric configuration and the glycosylated position at the constituent fructose moieties has seriously hampered isolation and structural characterization of pure compounds and, consequently, investigation of their individual properties. The interest in DFAs has experienced an explosion in the last two decades, fuelled by the identification of several representatives as components of the non-volatile fraction of aromatic caramels and chicory [18]. The implications of this discovery in the human diet have stimulated the development of new methods for their preparation. Reported methodologies involve both chemical and enzymatic approaches to transform affordable carbohydrate raw materials (inulin, sucrose, levan or fructose) into either controlled mixtures of DFAs or a particular DFA isomer. The chemistry of DFAs has been reviewed a decade ago [19]. This review is devoted to the recent advances in the stereoselective synthesis of DFAs by means of chemical and enzymatic approaches, their scope, limitations, and complementarities.

## 2. Chemical, Nutritional, and Technological Interest of DFAs

Besides its traditional involvement in the preparation of homemade products such as pastries, caramelization is nowadays an important industrial process used for the production of food materials and colouring additives, mainly from sucrose but also from d-glucose, maltose, maltodextrins and starch syrups [20,21]. Products having various properties are commercially available, depending on the starting material and the temperature and duration of the heating process. Food grade acids are frequently used as caramelization promoters. In any case, caramelization results in pH lowering, which self-catalyses the caramelization process. Protonic activation of the sugar precursor then occurs, leading to formation of volatiles (e.g. 2-hydromethylfurfural, HMF), coloured polymers (melanoidins) and a major fraction of oligosaccharidic nature whose structure remained unknown until recent times [18,19,20,21].

**Figure 1:**
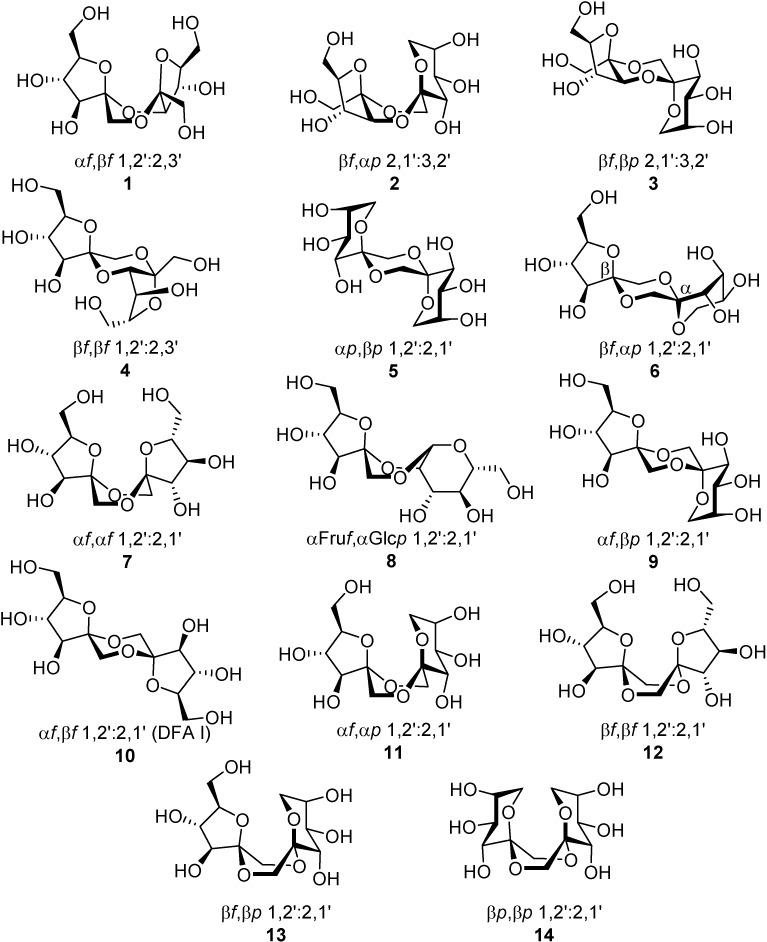
DFA structures identified in the disaccharide fraction of fructose and sucrose caramel.

DFAs are formed upon thermal or protonic activation of fructose and fructose-containing disaccharides (sucrose) and oligosaccharides (levan and inulin), which includes caramelization of these raw materials. Under such conditions, a fructosyloxacarbenium cation is generated, which undergoes *in situ* glycosylation by reacting with a second fructose unit. A transient ketodisaccharide is then formed, which undergoes intramolecular spiroketalization to furnish the DFA tricyclic core. A complex distribution of isomers that differ in the ring size, linking position, and stereochemistry at the ketal stereocenters is generally obtained. For instance, up to five different tricyclic cores and 13 DFA isomers have been identified in the disaccharidic fraction of fructose caramel (Figure 1). An additional mixed dianhydride containing a glucose subunit [α-d-fructofuranose-α-d-glucopyranose-1,2’:2,1’- dianhydride, (**8**)] has been identified in the disaccharidic fraction of sucrose caramel.

**Figure 2:**
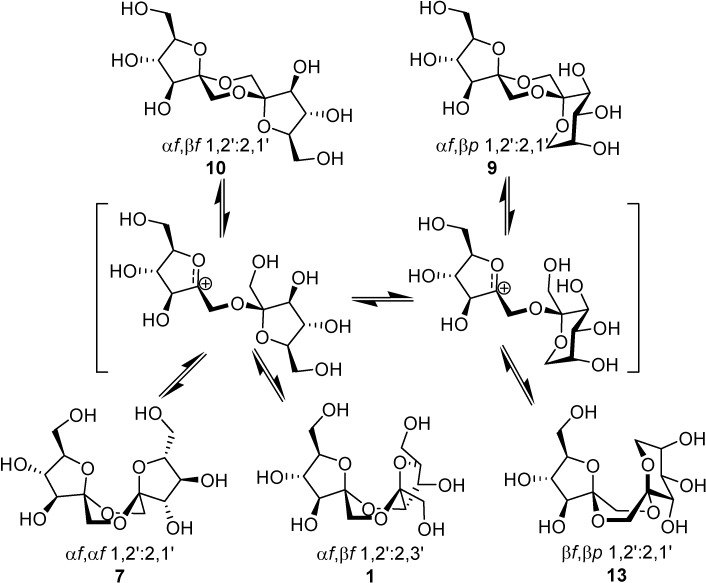
DFA isomerization reactions.

Spiroketalization is a reversible process under normal (thermal or protonic) caramelization conditions. Consequently, the isomeric distribution of DFAs in the mixture can be rationalized in terms of kinetic and thermodynamic control. Therefore, as observed by Manley-Harris and Richards for inulin pyrolysis [21,22], the relative abundance of isomeric DFAs is a balance between their rate of formation and disappearance. Dispiro-difuranose isomers [e.g., di-α-d-fructofuranose-1,2’:2,1’- dianhydride (**7**) or α-d-fructofuranose-β-d-fructofuranose-1,2’:2,1’-dianhydride (**10**)] are more abundant when short reaction times are applied, while longer reaction times favor the thermodynamically more stable pyranose isomers [e.g., α-d-fructofuranose-β-d-fructopyranose- 1,2’:2,1’-dianhydride (**9**)] and monospiro compounds [e.g., α-d-fructofuranose-β-d-fructofuranose- 1,2’:2,3’-dianhydride (**1**)] (Figure 2).

Since DFA formation is associated with heating of carbohydrate materials, DFAs are potentially present in any carbohydrate-rich foodstuff that has been subjected to cooking or that contains caramel or another cooked sugar as an additive. Actually, DFAs have been postulated as chemical markers and tracers of some food products. Defaye and co-workers have developed a GC-based analytical method that unequivocally assesses the authenticity and origin of caramels by observing the DFA fraction pattern in the chromatogram [23]. The same procedure has been applied to detect honey adulteration with commercial syrups [24] and to differentiate between natural- and sugar-roasted torrefacto coffee [25].

**Figure 3:**
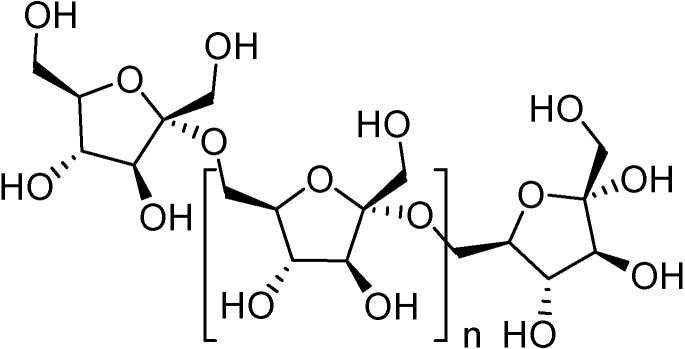
Structure of FOS.

Nutritional studies on DFAs reveal some similarities between this family of cyclic fructodisaccharides and the reducing fructooligosaccharides (FOS) used as prebiotics in the food industry. FOS are mixtures of linear oligosaccharides containing five to ten β-(2→1)-linked fructose units (Figure 3). They are non-digestible substances and behave as soluble alimentary fibers, facilitating intestinal motility. In mammals, including humans, FOS are known to exert a well studied bifidogenic effect in the colon [26,27] and are therefore extensively used as food additives [28,29].

In most of the patent files dealing with DFAs, they are rated as low-caloric sweeteners. Bifidogenic, anticariogenic and anti-tooth decaying effects have been also claimed. Moreover, DFAs promote *in vitro* growth of bifidobacteria [30]. Some of the benefits associated to a healthy *Bifidobacteria* spp. population in the digestive tract of mammals are carcinogenesis inhibition [31], the decrease of blood pressure and blood cholesterol levels [32], vitamin B-complex synthesis stimulation [33] or inhibition of proliferation of undesirable bacteria such as *Clostridium perfringens* or *Escherichia coli*, among others [34,35]. Some studies have revealed that the use of DFA-containing products in animal feeding (i.e., fowls) protect against digestive tract infections [36]. These prebiotic effects are the basis of several inventions, like that described by Stoppok *et al*. consisting of a beverage containing DFAs as dietary fiber [37].

**Figure 4:**
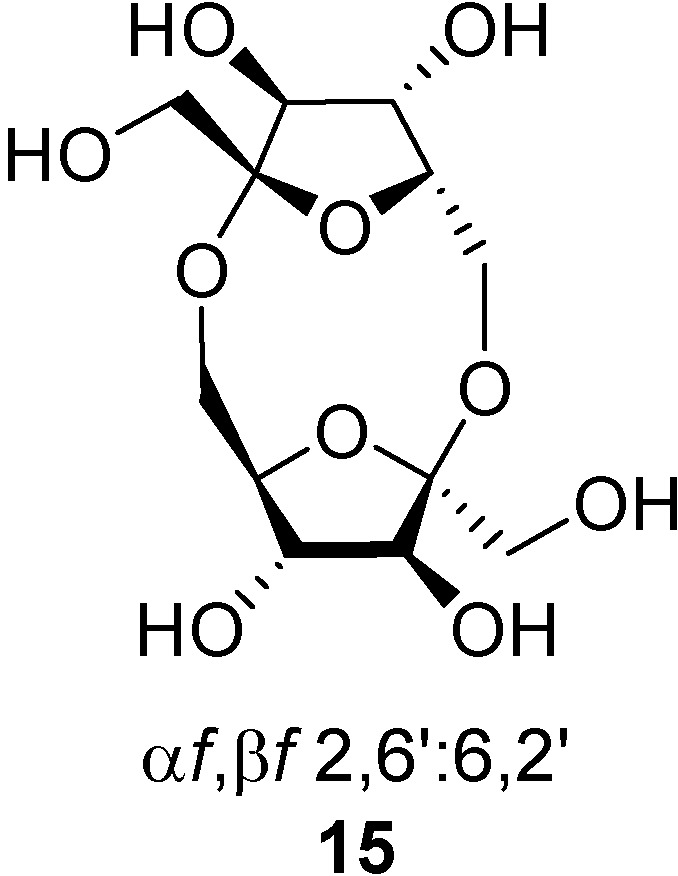
Structure of the non-spiroacetalic di-β-d-fructofuranose 2,6’:6,2’ dianhydride (**15**).

Several reports state that certain DFAs isomers are involved in intestinal metal cation absorption. For instance, Hara and Tomita have demonstrated that ingestion of α-d-fructofuranose-β-d-fructofuranose 1,2’:2,3’-dianhydride (**1**, also known as DFA III) in daily diet increases the absorption of calcium, magnesium and zinc in rats [38,39,40,41,42,43]. The same authors observed that ingestion of DFA III and DFA IV [di-β-d-fructofuranose-2,6’:6,2’-dianhydride (**15**), a non-spiroacetalic DFA isomer, Figure 4] [44] affects the epithelial tissue and activates the passage of tight junctions in vitro, thereby promoting mineral absorption in the small and large intestine of rats [45,46]. α-d-Fructofuranose β-d-fructofuranose-1,2’:2,3’-dianhydride (**1**) also prevents tannic acid-induced suppression of iron absorption and contributes to bone strength, thereby protecting against anaemia [47] and preventing osteoporosis [48]. Interestingly, these properties are characteristic of certain DFAs and have not been observed in other fructooligosaccharides.

Very recently, preliminary results concerning the effects of DFAs in the human diet have been reported. DFA III, one of the very few isomers available at technical scale, increases iron absorption and retention in female college students [49] and a similar effect has been reported for calcium in healthy men [50].

**Figure 5:**
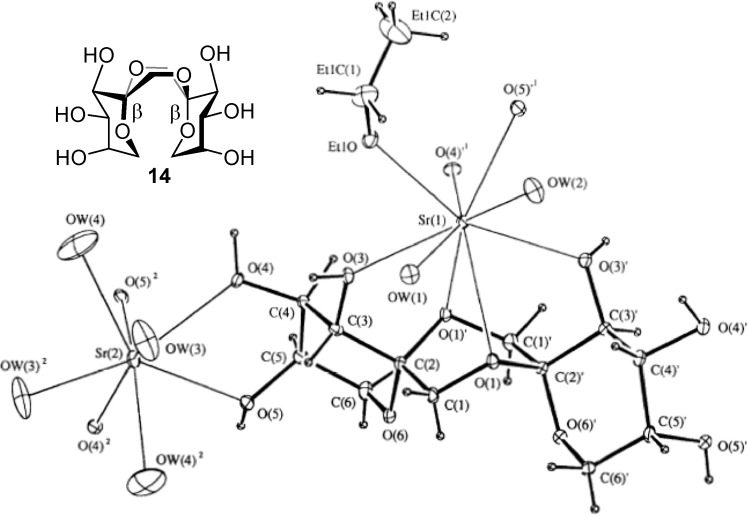
X-Ray structure of the Sr^2+^−di-β-fructopyranose-1,2’:2,1’-dianhydride (**14**) complex (reproduced from reference [53], with permission of NRC Research Press).

From a technical point of view, other than nutritional, the most interesting features of DFAs are probably the high rigidity of their structure and their hydrophilicity, which is shared with many sucrochemicals [51,52]. These characteristics can be exploited, for instance, in the preparation of surfactants, hydrophilic polymers, or complexing agents, to mention just a few. Indeed, *C*_2_-symmetric d-fructose-1,2’:2,1’-dianhydrides have been reported to form complexes with metal cations such as Ca^2+^ and Sr^2+^ [53]. A conformational study carried out by Pedersen and coworkers revealed that a boat conformation in the central 1,4-dioxane ring is predominant in these isomers due to the interplay of the anomeric effect at the spiroketal centers and the exo-anomeric effect related to the fructose moieties [54]. The boat (or skew-boat) conformer is the only one detected both in the solid state and in solution for di-β-d-fructopyranose-1,2’:2,1’-dianhydride (**14**), for instance [53]. This arrangement allows both dioxane oxygen atoms to coordinate to a metal cation simultaneously and, at the same time, fixes a favorable orientation of the hydroxyl groups to participate in complex stabilization (Figure 5). It has been postulated that such cation complexing DFAs might find an application as detergent builders or co-builders because of their ion-chelating and sequestering properties, representing an alternative to the banned tripolyphosphates [55].

**Figure 6:**
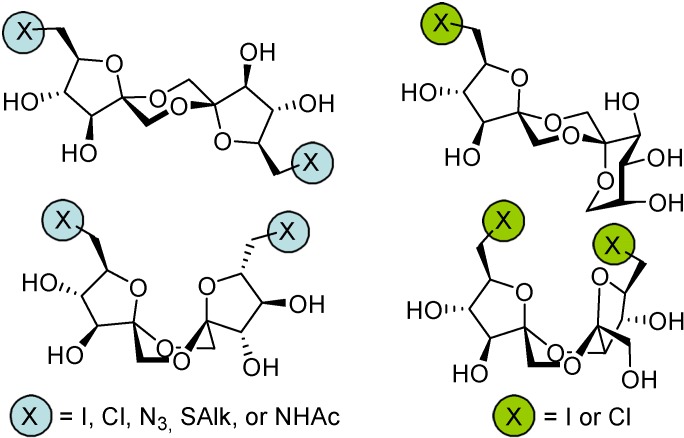
DFA derivative libraries [56].

DFAs are also attractive synthetic intermediates. Their stability towards heat and acid hydrolysis makes them compatible with a wide range of reaction conditions. Applications as building blocks or scaffolds for chemical synthesis have been outlined. For instance, taking advantage of reported strategies for the selective functionalization of sucrose and fructooligosaccharides, García Fernández and Defaye have reported the preparation of libraries of DFA derivatives bearing diverse functional groups on their primary positions (i. e. halogens, azide, thioether, amine, or amide) (Figure 6) [56]. Some of the members of these libraries have shown remarkable properties as liquid crystals, while others were excellent precursors of hydrophilic polymers or non-cationic surfactants.

A much greater body of knowledge must be gained about the effects of ingesting the different isomeric DFAs. The lack of efficient production and purification methodologies to obtain pure DFA isomers has been a major limitation for such goal. During the last decade, major advances concerning the stereoselective synthesis of DFAs have been achieved that may significantly change the current status of this kind of spirocompounds in carbohydrate chemistry and biology. The increasing interest of the food industry in developing new “functional products” is motivating pure academic research in the field, which certainly will bring about new applications for this family of carbohydrate spiroketals.

Expectation for commercial viability of DFAs is further sustained by the fact that the raw materials from which they can be obtained are readily available from comparatively inexpensive agricultural feedstocks. DFA-producing oligosaccharides such as inulin or levan (consisting of β2→1 and β2→6-linked fructosides, respectively) are abundant starting materials. They are energy storage carbohydrates in higher plants, such as Jerusalem artichoke or chicory for the former and ryegrass or cocksfoot for the latter. Chicory, in particular, might eventually be considered to replace sugar beet in certain geographical areas where climate restrictions hampers the cultivation of the latter. Chicory tolerates cold, diseases and desiccation, while crop yields are much higher than those for corn or sugar beet (up to 45 tons per ha) [57,58]. Efficient large scale preparation of these oligosaccharides using sucrose industry technologies has been demonstrated. For instance, inulin has been produced in yields up to 46%, which compares very favorably with the 20-25% yield of sucrose obtained from sugar beet [59].

## 3. Chemical Strategies towards the Stereoselective Synthesis of DFAs

### 3.1. Synthesis of DFAs by protic acid activation of unprotected d-fructose precursors

**Scheme 1:**
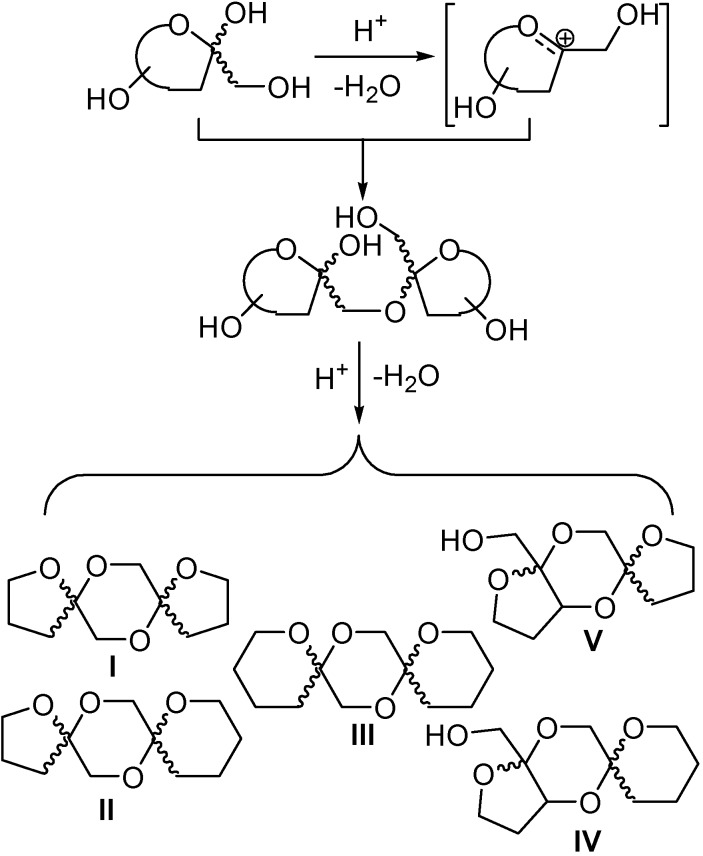
Protic acid-catalyzed dimerization of ketoses.

Despite the variety of synthetic methods existing for the stereoselective construction of the spiroketal moiety [1,2,3,4,5], the main strategy still relies on the acid-catalyzed intramolecular cyclization of the corresponding dihydroxyketo precursors or their equivalents. The stereochemical outcome of this transformation is controlled, almost exclusively, by the relative thermodynamic stability of the different isomers. When all factors that control spiroketalization, that is, a maximum anomeric effect and minimum steric interactions, are reinforcing, a major isomer is produced. In the case of bis(spiroketal) derivatives, and for DFAs in particular, a range of structures can usually accommodate the basic requirements, namely oxygen subtituents at anomeric centres in axial disposition and carbon substituents in equatorial orientation, with rather small difference in energy and low interconversion barriers. Up to five different mono and dispiroketalic tricyclic cores (i. e. types I to V) have been so far identified in reaction mixtures from the protic acid-catalyzed dimerization of d-fructose. These cores are classified as types I-V where, for instance, type I refers to 1,2’:2,1’-linked difuranose DFA isomers or type IV refers to 1,2’:2,3’-linked furanose pyranose DFAs (Scheme 1). Although their relative proportions can be varied, to some extent, by modulation of the acid strength and reaction conditions (temperature, reaction time and initial concentration), isolation of pure samples from these isomeric mixtures remains problematic [15,18,19].

The accepted mechanism for DFA formation involves a fructosyl oxacarbenium cation that undergoes *in situ* glycosylation to the corresponding keto-disaccharide by reacting with a second fructose molecule. Further intramolecular spiroketalization to close the central 1,4-dioxane ring is a reversible process, the ratio of the different isomeric products varying with time, temperature, and initial concentration. In some favourable cases, the reaction can be driven to the preferential formation of a particular DFA isomer [56].

**Scheme 2:**
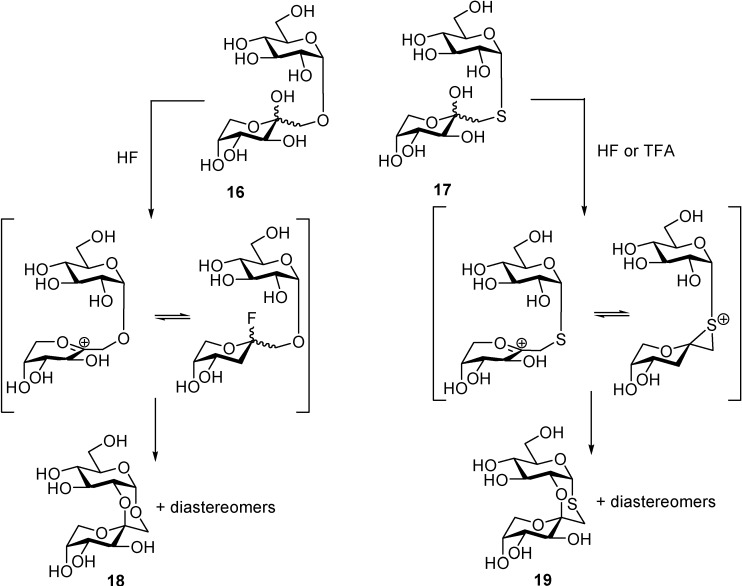
Comparative protonic-promoted spiroketalization of trehalulose and thiotrehalulose.

High-yielding preparations of DFAs have been previously achieved by protonic activation of d-fructose, fructooligosaccharides, sucrose or inulin with anydrous hydrogen fluoride (HF) or its complex with pyridine [60,61,62]. HF is an excellent solvent for carbohydrates and a non-dehydrating, strong protonating reagent capable to activate selectively the anomeric position of the sugar precursor. In certain circumstances, the fructosyl oxacarbenium intermediate might be in equilibrium with the corresponding glycosyl fluoride, itself a powerful glycosyl donor [63]. The use of HF has been successfully extended to promote spiroketal formation also from fructose-derived thiodisaccharides such as 1’-thiotrehalulose (**17**) [64]. In this case, a much higher intramolecular spiroketalization rate was observed as compared with the corresponding glucosylfructose trehalulose (**16**). Spiroketalization could be efficiently promoted even by using much less strenuous conditions, e.g. 9:1 trifluoroacetic acid-water, supporting the involment of transient episulfonium intermediates at the anomeric position, instead of the fructosyl fluoride, as the active species (Scheme 2).

### 3.2. Stereoselective synthesis of DFAs by ring-size blocking of the ketose precursor

The protonic activation approach for the preparation of DFAs presents, however, serious limitations when seeking preparation of individual diastereomers. Under these conditions, spiroketalization is a fully reversible process. Consequently, the composition of the mixture is dependent not only on the starting cyclic form of the d-fructose moiety in the d-fructose-containing precursor [5], but also on the tautomeric equilibria at both fructose subunits in the dimer and the presence of several hydroxyl groups liable to participation in spiroketal ring closing. Furthermore, this strategy precludes the possibility of an efficient preparation of interesting DFA isomers, such as di-β-d-fructopyranose-1,2’:2,1’- dianhydride (**14**), which are neither the kinetic nor the thermodynamic product as a consequence of stereoelectronic considerations. As commented previously, in these *C*_2_-symmetric derivatives, the dispirodioxane ring must adopt a boat conformation in order to comply with both, the anomeric and exoanomeric effects [53]. Such conformation, which is favourable for cation complex formation, is far higher in energy as compared with other isomeric DFAs.

**Scheme 3:**
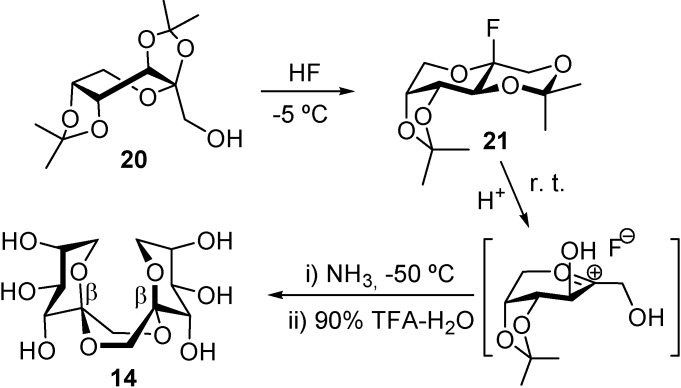
Hydrogen fluoride-promoted stereoselective synthesis of di-d-β fructopyranose-1,2’:2,1’-dianhydride (**14**).

The weaknesses of the above strategy have been tackled by implementing methodologies that prevent reversion during DFA formation in combination with the use of protecting groups to anchor the furanose or pyranose cyclic form. This notion was first exemplified by García Fernández and Defaye [65] for the stereospecific synthesis of DFA **14** [53]. Activation of 2,3:4,5-di-*O*-isopropylidene-β-d-fructopyranose (**20**), readily accessible in one step from commercial d-fructose [66], with anhydrous HF resulted in the rearrangement of the anomeric isopropylidene group, with simultaneous formation of a reactive β-fructopyranosyl fluoride (**21**). Upon neutralization of the reaction mixture with ammonia, **21** underwent dimerization to give the target *C*_2_-symmetric DFA in 60% overall yield as the only reaction product (Scheme 3).

The efficiency of the above transformation deserves a further comment. The presence of the cyclic acetal group at O-4 and O-5 in the starting d-fructose derivative is not only preserving the pyranose cyclic form during spiroketalization, but also stereodirecting the reaction course to the formation of the β-anomeric linkages. The authors state that the effect is probably related to steric hindrance at the α-side in the intermediate oxocarbenium ion, due to the presence of the bulky isopropylidene group, as well as to destabilisation of the known ^5^*C*_2_ conformation of the α-fructopyranose subunit in the isomeric α-d-fructopyranose β-d-fructopyranose 1,2’:2,1’-dianhydride [60,61,62].

Though hydrogen fluoride itself is a mild acid promoter, the harsh working conditions involved in handling HF and ammonia prevented the extension of this approach to the selective synthesis of other DFA isomers. A more user-friendly methodology to achieve activation of *O*-protected d-fructose precursors bearing an anomeric cyclic acetal group has been reported by Ortiz Mellet and García Fernández. Their methodology relies on the ability of selectively protected 1,2-*O*-isopropylidene-β-d-fructofuranose and fructopyranose derivatives to undergo a tandem transformation upon treatment with Lewis acids in non-polar solvents, involving (i) acetal cleavage, (ii) intermolecular glycosylation, and (iii) intramolecular spiroketalization [67].

**Scheme 4:**
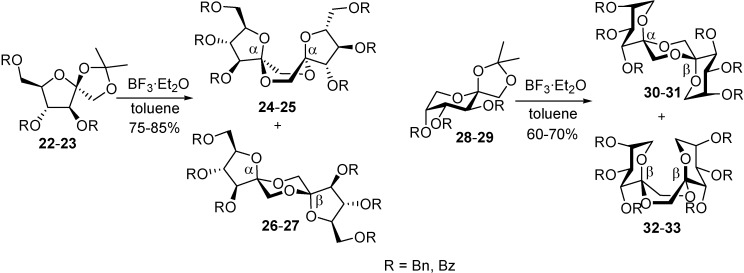
Boron trifluoride-promoted stereoselective synthesis of type I and III DFAs.

In contrast to the aqueous mineral acid-catalyzed reaction, spiroketalization in apolar organic solvents occurs under irreversible conditions, thus limiting isomerization processes. By using boron trifluoride-diethyl ether complex, a Lewis acid widely used for the cleavage of acetal protecting groups [68], as promoter in glycosylation reactions [69] and as catalyst in spiroketalization processes [5], efficient one-pot syntheses of difructofuranose (type I, Scheme 4 right) and difructopyranose (type III, Scheme 4 left) dianhydrides were performed.

**Table 1:** Boron trifluoride-promoted DFA synthesis (n.d., not detected).

Starting material	R	Products (relative proportion)		
		α,α	α,β	β,β
**22**	Bn	**24**	**26**	n.d.
		(1 : 2.5)		
**23**	Bz	**25**	**27**	n.d.
		(25 : 1)		
**28**	Bn	n.d.	**30**	**32**
			(25 : 1)	
**29**	Bz	n.d.	**31**	**33**
			(1 : 1)	

**Figure 7:**
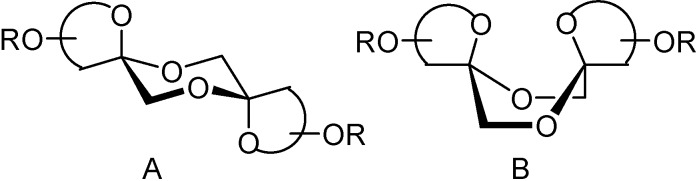
Conformations at the central dioxane ring for non-symmetric (A) and *C*_2_-symmetric (B) DFAs.

The possibility to control not only the ring size but also the stereochemistry at the spiroketal centers by using participating (ester) or not participating (ether) protecting groups is noteworthy (Table 1). The preference for dissymmetric dispiroketal structures in the case of nonparticipating benzyl protecting groups (Scheme 4, α,β isomers) is in agreement with reported observations for other spiro-ketooligosaccharides [70,71]. These diastereomers can accommodate the anomeric effect at both spiroketal centres with the central 1,4-dioxane ring in a chair conformation being thermodynamically favoured. In order to comply with the anomeric effect, DFAs having identical configuration at both spiroketal centres must adopt instead a boat conformation, an unfavourable arrangement (Figure 7).

**Figure 8:**
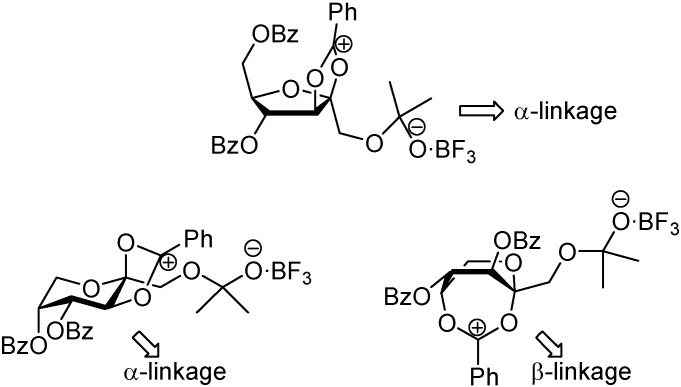
Probable structures of the acyloxonium cations involved in the dimerization reaction of acylated d-fructose precursors.

In the case of participating protecting groups, the authors propose a reaction mechanism involving acyloxonium intermediates. In the furanose series, the formation of a 2,3-acyloxonium species would block the β-face of the monosaccharide, favouring the formation of the *C*_2_-symmetric α,α-isomer. In fructopyranose derivatives, the 2,3-acyloxonium is probably in equilibrium with a 2,5-acyloxonium intermediate. While the first one would direct glycosylation and spiroketalization to form α-linkages, the second would lead to the opposite anomer. As a result, a 1:1 mixture of the dissymmetric (α,β) and *C*_2_-symmetric (β,β) diastereomers is obtained (Figure 8).

The use of anhydrous trifluoromethanesulfonic acid (triflic acid) as promotor has been shown to increase the overall yield in the above transformations, though diasteroselectivity was not significantly improved [72]. A catalytic version of the procedure have been recently described [73].

### 3.3. Stereoselective synthesis of DFAs by conformational control of fructosyl donors

**Figure 9:**
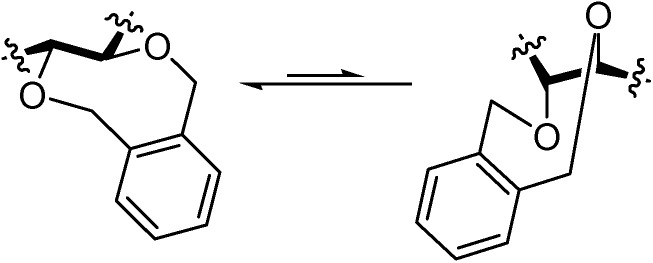
Schematic representation of *o*-xylylene-protected *trans*-diequatorial (left) and trans-diaxial (right) 1,2-diol segments.

The observation of the influence exerted by protecting groups on the stereochemical outcome of spiroketalization reactions has led to new strategies to improve selectivity in the synthesis of DFAs. Balbuena *et al*. [74] have designed monomeric d-fructose precursors with predictable conformational biases for stereochemical control during spiroketalization. Their strategy relies upon the use of the cyclic *o*-xylylene group, recently introduced for benzyl-type protection of *vic*-diols in carbohydrate chemistry [75,76], to preserve diequatorial, in opposition to diaxial, dispositions (Figure 9).

**Figure 10:**
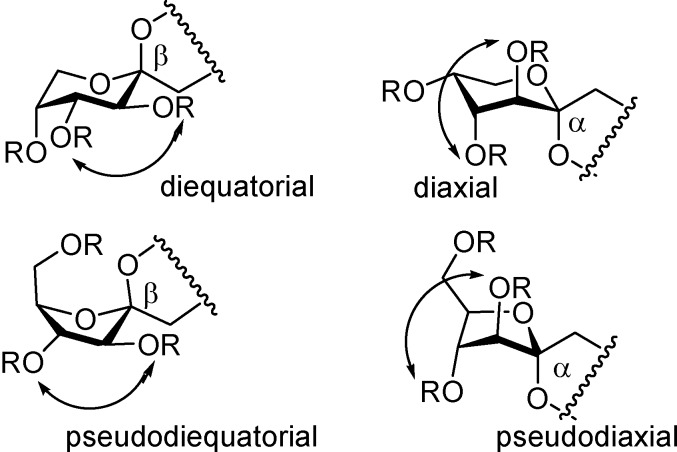
Preferred conformations of the fructopyranose (top) and fructofuranose rings (down) in dispirodisaccharides. The relative disposition of the *trans*-oriented substituents at C-3 and C-4 is indicated.

**Scheme 5:**
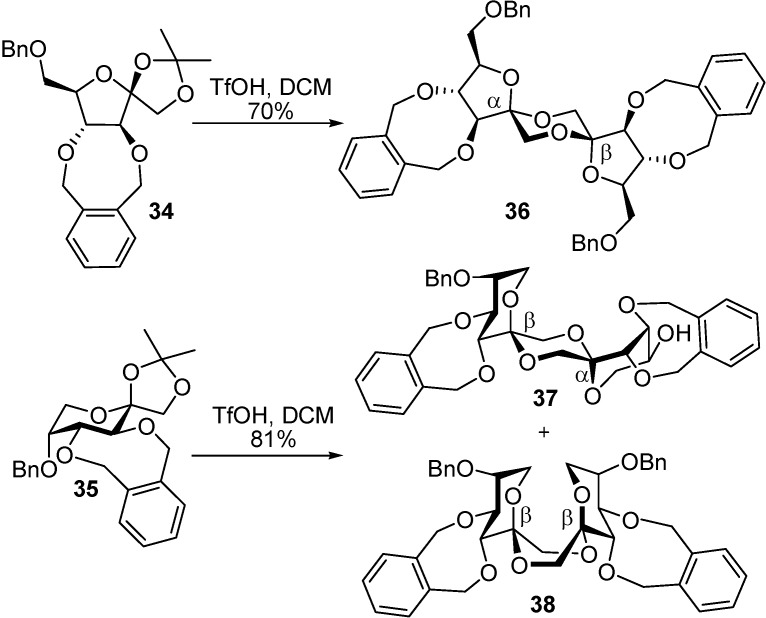
Stereoselective synthesis of type I and III DFAs from *o*-xylylene protected precursors.

A comparative analysis of the conformational behaviour of the fructose moieties in dispirodisaccharides revealed that in α-configured rings the hydroxyl groups at C-3 and C-4 adopt a diaxial (or pseudodiaxial) disposition, while in the β-configured counterparts they prefer a diequatorial (or pseudodiequatorial) orientation (Figure 10). The insertion of a cyclic *o*-xylylene group at this segment allows the remote control of the stereochemistry during spirocyclization. Thus, triflic acid-promoted dimerization of the furanose derivative **34** afforded exclusively the α,β type I DFA **36**, while the pyranose derivative **35** afforded a 3:1 (β,β:α,β) mixture of the type III DFAs **38** and **37** (Scheme 5).

Comparison of the results obtained for tri-*O*-benzyl precursors (Scheme 4 and Table 1) with those using the cyclic xylylene protection clearly demonstrates the stereodirecting effect of the *o*-xylylene group in bis-spiroketal-forming reactions (Table 2). In the furanose series, the isomer presenting a trans-diequatorial diol segment (α,β) is also thermodynamically favoured, resulting in total stereoselectivity in the creation of the two spiroketal centres. For the pyranose counterparts, an over 100-fold increase in the selectivity of the reaction towards the *o*-xylylene-controlled β,β-diastereomer **38** was attained by replacing the O-3 and O-4 benzyl groups with a cyclic *o*-xylylene group (the α,β: β,β ratio shifts from 25:1 to 1:3).

**Table 2:** Comparative results for DFA formation from d-fructose precursors bearing acyclic and cyclic benzyl-type protecting groups (n.d., not detected).

Starting material	Products (relative proportion)		
	α,α	α,β	β,β
**22**	**24**	**26**	n.d.
	(1 : 2.5)		
**34**	n.d.	**36**	n.d.
**28**	n.d.	**30**	**32**
		(25 : 1)	
**35**	n.d.	**37**	**38**
		(1 : 3)	

### 3.4. Stereoselective synthesis of DFAs via intramolecular aglycon delivery

The concept of *intramolecular aglycon delivery* (IAD) [77], introduced by Hindsgaul [78,79,80] and Stork [81,82] in oligosaccharide synthesis, has been extended to the stereoselective preparation of DFA isomers that are neither thermodynamically nor kinetically favoured (contra-thermodynamic [83,84,85] DFAs). The approach takes advantage of the rigidity of the tricyclic bis(spiroketal) structure and the dependence of the conformational properties on the relative configuration at the spiroketal centres. This is translated into significant differences in the inter-space distance between homologous hydroxyl groups located at the two d-fructose moieties in the DFA molecule within an isomeric series. Inserting a distance restriction element between appropriate positions can then be used to introduce a geometrical constraint during spirocyclization.

**Figure 11:**
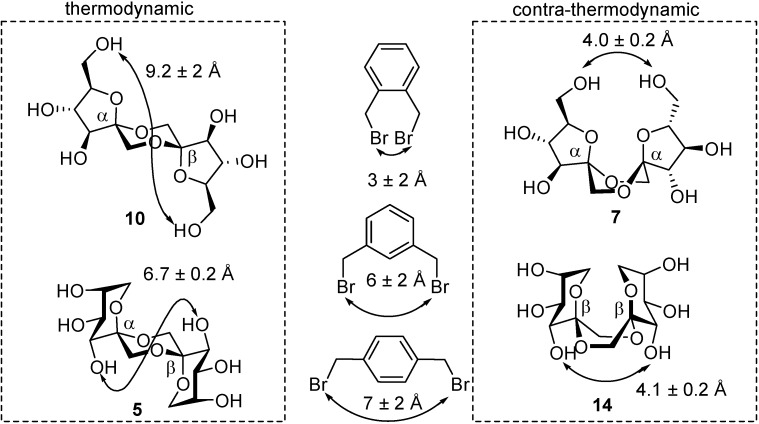
Preferred conformations of type I and type III DFAs positional isomers, with indication of the O-6−O-6', O-3−O-3' interatomic distances, respectively. The distances between the benzylic methylene carbon atoms in α,α'-dibromoxylene positional isomers are also shown.

To support the above hypothesis, the incorporation of xylylene positional isomers as tethers to pre-define a given separation between selected positions during spiroketalization has been put forward. This strategy is inspired by the “rigid spacer-mediated linkage via non-reacting centers” concept, previously exploited by Schmidt and coworkers in anomeric configuration control during glycosidic bond-forming reactions [86,87,88]. The possibility to finely tune the distance and flexibility by using commercially available α,α’-dibromo-*o*-, -*m*- or -*p*-xylene as tethering reagents, offers a unique opportunity to control the stereochemical outcome of the intramolecular transformation. This approach has been investigated in depth for the preparation of bis(spiroketal) difuranose (type I) and dipyranose (type III) DFAs (Figure 11) [89,90].

**Scheme 6:**
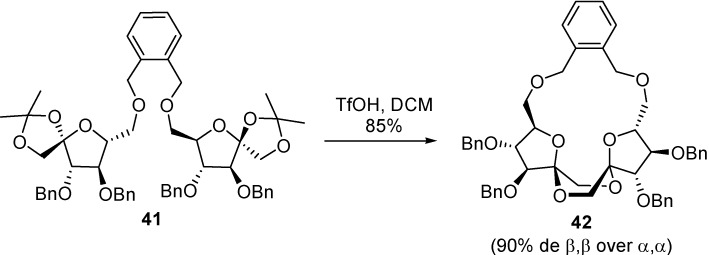
Stereoselective synthesis of type I DFAs by IAD using the *o*-xylylene tether. Similar transformations from *m*- and *p*-xylylene tethered precursors have been studied (see Table 3).

**Scheme 7:**
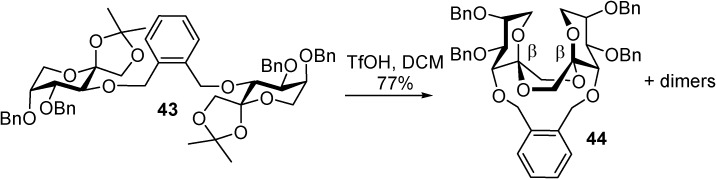
Stereoselective synthesis of type III DFAs by IAD using the *o*-xylylene tether. Similar transformations from *m*- and *p*-xylylene tethered precursors have been studied (see Table 4).

Considering the much shorter distance between the primary O-6 oxygens in the case of *C*_2_-symmetric difuranose DFAs, or between the O-3 oxygens in the case of *C*_2_-symmetric dipyranose DFAs, it was conceivable that the shortest *o*-xylylene tether would improve the stereocontrol for the contra-thermodynamic isomers upon spiroketalization, while the more flexible *m*-xylylene bridge should result in increased proportions of the thermodynamic counterparts. The hypothesis was validated both for the stereoselective synthesis of contra-thermodynamic type I (Scheme 6, Table 3) and type III (Scheme 7, Table 4) DFA isomers **42** and **44**, respectively [89,90].

**Table 3:** DFA formation from *o*-, *m*- and *p*-xylylene-bridged d-fructofuranose precursors (n.d., not detected).

Xylylene tether	Product, yield			
	α,α	α,β	β,β	dimers
*o* (**41**)	8%	n.d.	**42**	6%
			71%	
*m*	50%	25%	n.d.	16%
*p*	12%	n.d.	3%	68%

**Table 4:** DFA formation from *o*-, *m*- and *p*-xylylene-bridged d-fructofuranose precursors. (n.d., not detected).

Xylylene tether	Product, yield		
	α,β	β,β	dimers
*o* (**43**)	n.d.	**44**	35%
		42%	
*m*	12%	48%	24%
*p*	n.d.	n.d.	**45**
			72%

**Figure 12:**
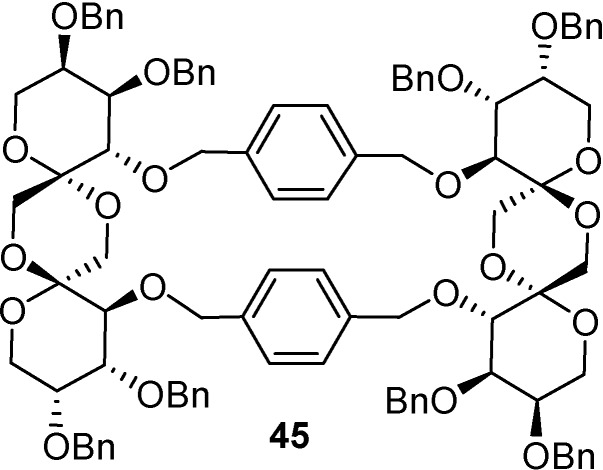
Structure of the *p*-xylylene-DFA macrocycle **45**.

The longer and less flexible *p*-xylylene tether favoured the formation of dimeric macrocyclic derivatives resulting from double bis-spirocyclization [90]. Although the dimer fraction was a mixture of all diastereomeric possibilities, the di-β-d-fructofuranose-1,2’.2,1’-dianhydride was the preferred substructure in the difructopyranose (type III) series (e.g., **45**, Figure 12), in agreement with preliminary calculations indicating the preference for macrocyclic *C*_2_-symmetric structures. Interestingly, the central dioxane ring in the DFA moieties in the *p*-xylylene-derived macrocyle **45** was not in the boat conformation expected for *C*_2_-symmetric DFAs, but in a distorted chair conformation, as seen by single-crystal X-ray diffraction [90]. This result suggests that contra-thermodynamic DFAs are probably more flexible than the thermodynamic isomers, being able to better accommodate conformational constraints.

Fixing the ring size in the fructose precursor has proven very successful for the stereoselective synthesis of DFAs of types I (difuranose) and III (dipyranose). Construction of the mixed furanose-pyranose DFA core (type II DFAs) core is, however, more difficult. Since the four diastereomeric possibilities with a d-fructofuranose d-fructofuranose-1,2’:2,1’-dianhydride structure (**6**, **9**, **11** and **13**) are present in sucrose caramel, accounting for more than 20% of the disaccharide material, their preparation represents an important goal in order to provide access to pure DFA standards for analytical and nutritional studies.

**Figure 13:**
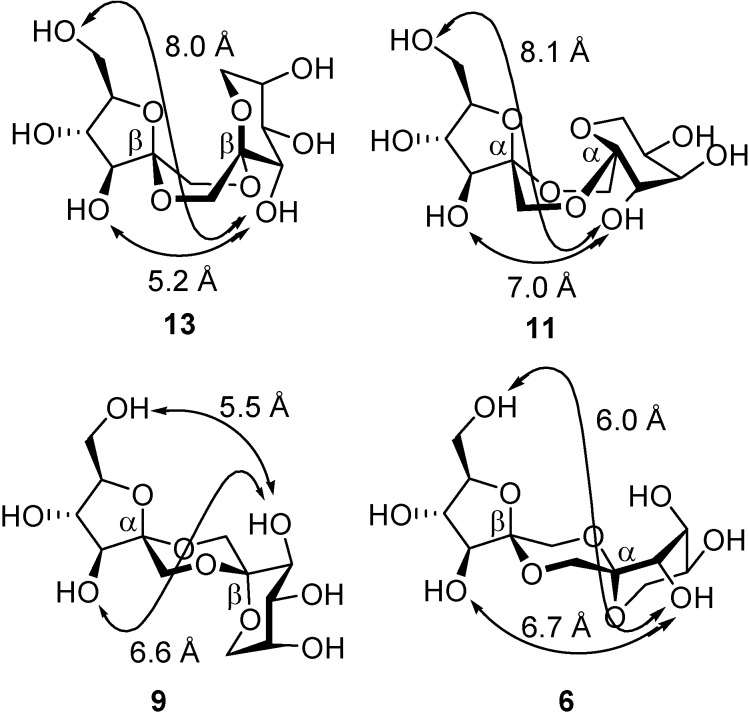
Conformations of the different type II DFAs and their O-3−O-3’ and O-6−O-3’ interatomic distances.

**Scheme 8:**
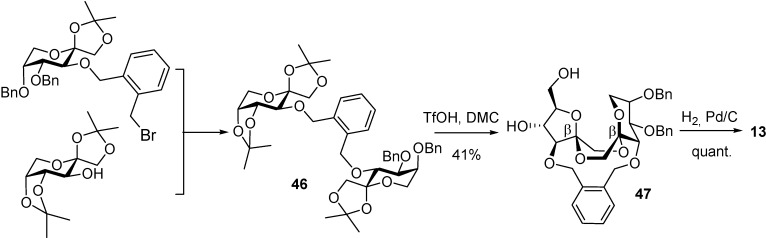
Spacer-mediated stereoselective synthesis of type II DFAs.

Rubio *et al*. [91] have developed a stereoselective synthesis of the β,β-type II DFA **13** by taking advantage of two considerations: (a) the significantly shorter O-3–O-3’ interatomic distance in this particular DFA derivative as compared with the other three diastereomers in the series (Figure 13), and (b) five-membered-ring spiroketals are kinetically favoured over six-membered rings. The methodology involves the *o*-xylylene tethering reaction of two differently protected difructopyranose derivatives to limit the conformational space during the intramolecular glycosylation-spirocyclization reaction (**46**). In one of them, the integrity of the six-membered ring is maintained during acid activation by the presence of a benzyl group at O-5, while in the other one, the labile isopropylidene group at this position is cleaved under the reaction conditions. The higher reactivity of the fructofuranosyl oxacarbenium cation as compared with the homologous six-membered isomer resulted in the formation of the furanose-pyranose DFA **47** as the only detectable bis-spirodisaccharide product (Scheme 8).

**Scheme 9:**
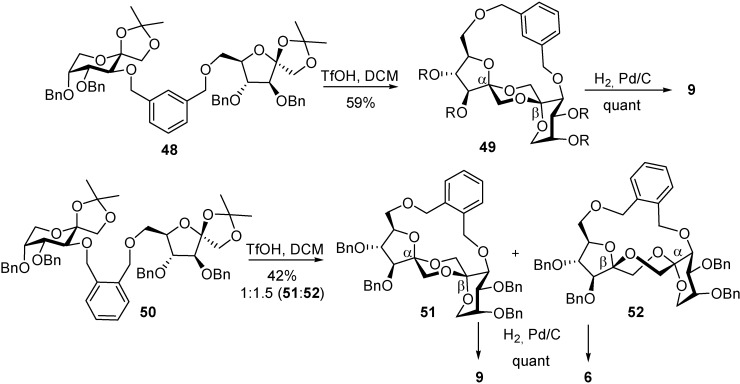
Spacer-mediated stereoselective synthesis of type II DFAs.

Louis *et al*.[92] have broadened the concept of rigid-spacer-mediated spiroketalization by taking advantage not only of distance restrictions but also of subtle molecular flexibility differences in the tricyclic bis(spiroketal) skeleton. The combination of both aspects have provided efficient synthesis for two additional type II DFAs, namely the α,β (**9**), a major constituent of commercial caramel (18% of the disaccharidic fraction), and the β,α (**6**) isomers. The authors realized that the primary position O-6 in the furanose moiety and position O-3’ in the pyranose ring lie much closer in the chair conformers than in the boat DFAs (Figure 13). Depending on the linker length and flexibility, the reaction proceeds preferentially either intra- (*o*- and *m*-xylylene bridges) or intermolecularly (*p*-xylylene tether; Scheme 9). For the more flexible *m*-xylylene positional isomer (see precursor **48**), the intramolecular reaction led exclusively (59% yield) to the thermodynamic α-fructofuranose-β-fructopyranose-1,2’:2,1’-dianhydride derivative **49**. The shorter *o*-xylylene tether (see precursor **50**) afforded preferentially the elusive contra-thermodynamic β-fructofuranose α-fructopyranose diastereomer **52**, in addition to the α,β isomer **51** (42% yield, **51**:**52** relative proportion 1:1.5) [93].

The remarkable switch in the stereochemical outcome effected by the spacer length is attributed to the above-mentioned higher flexibility of contra-thermodynamic DFAs as compared to thermodynamic isomers [90]. While the rigid α,β derivative **51** can only partially alleviate steric stress by twisting the α-furanose ring, in the contra-thermodynamic isomer **52** the α-pyranose ring can flip from the ^5^*C*_2_ to the ^2^C_5_ chair. The loss of an anomeric effect interaction is compensated by twisting the central 1,4-dioxane ring to a skew-boat conformation to fully release steric constraints.

To sum up, the above commented strategies allow the stereoselective synthesis of bis(spiroketal) DFAs of type I (difuranose), type II (furanose-pyranose), and type III (dipyranose). In contrast, the stereoselective chemical synthesis of monospiroketal DFAs of types IV and V has not been achieved yet. Most of these DFA isomers are present as trace components in industrial caramels. Only the type V α-fructofuranose β-fructofuranose 1,2’:2,3’-dianhydride (**1**, also known as DFA III) represents a significant fraction in food products (up to 8% in sucrose caramel). Since some DFAs are produced by several microorganisms or even by higher plants, biotechnological methods can be an interesting alternative for their preparation. Actually, DFA III is nowadays easily available by enzymatic means on a technical scale. The advances in such enzymatic methods are discussed below.

## 4. Enzymatic Strategies towards the Synthesis of DFAs

The first evidence of specific DFA formation in higher plants dates back to 1933, when Schlubach and Knoop [94] isolated a compound tentatively identified as α-fructofuranose β-fructofuranose 1,2’:2,1’-dianhydride (**10**, also known as DFA I) from Jerusalem artichoke. The investigation of DFAs as natural products has been very limited until the mid-70s, when Tanaka and Uchiyama reported the isolation of an extracellular inulinase from *Arthrobacter ureafaciens*, a soil bacteria that synthesized α-fructofuranose β-fructofuranose 1,2’:2,3’-dianhydride (DFA III) from inulin [95,96].

**Table 5:** Nomenclature of DFA-producing enzymes.

EC 4.2.2.16	
common name	levan fructotransferase (DFA IV-forming)
product	di-β-D-fructofuranose-2,6':2',6-dianhydride(non spiroketalic DFA)
**EC 4.2.2.17** (formerly EC 2.4.1.200)	
common name	inulin fructotransferase (DFA I-forming)
product	α-D-fructofuranose β-D-fructofuranose-1,2':2,1'-dianhydride
**EC 4.2.2.18** (formerly EC 2.4.1.93)	
common name	inulin fructotransferase (DFA III-forming)
product	α-D-fructofuranose β-D-fructofuranose-1,2':2,3'-dianhydride

These seminal results have prompted much interest in the biosynthetic routes to DFAs, which however has been almost exclusively located in Japan. Unfortunately, a quantity of otherwise relevant papers was published in local journals, which limited their scope and accessibility. In a more general revision of the subject, Uchiyama has collected the most significant contributions to microbial biosynthesis and degradation of fructans up to 1993 [97]. In 2000 Saito and Tomita [98] reviewed particular issues concerning mass production of DFAs following biosynthetic routes and, very recently, Kawamura and Uchiyama have collated the recent advances in the enzymatic production of DFAs and cyclofructans [99].

**Figure 14:**
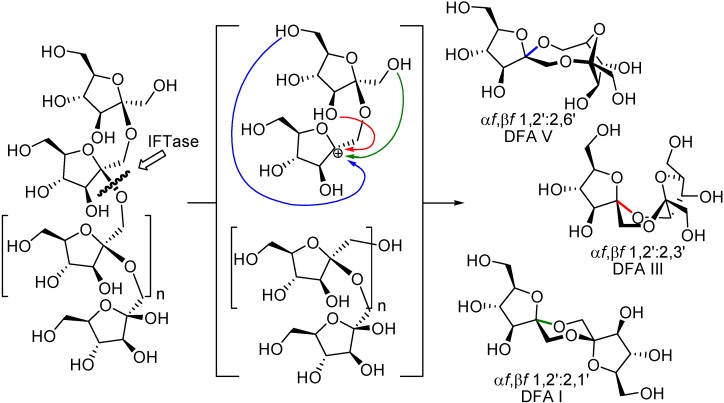
IFTase-catalyzed synthesis of DFAs.

Since Tanaka and Uchiyama first report on the isolation of a DFA III-forming enzyme, several microorganisms, many of which belong to the *Arthrobacter* genus, have been shown to produce enzymes that promote the transformation of fructans (inulin or levan) into DFAs (Table 5). As a unique feature, and in stark contrast with common fructan decomposing enzymes, these enzymes catalyze an intramolecular transglycosylation reaction through which the second glycosidic bond from the non-reducing fructose is transferred to the terminal residue, thus releasing a DFA molecule (Figure 14). This mechanism operates for both, inulin degrading (IFTase) [100] and levan degrading (LFTase) enzymes [101,102,103]. Though, IFTase have long been classified as “transferases”, they have been recently re-categorized as “lyases” (EC 4) since glycon transfer occurs intramolecularly through an elimination reaction [104].

**Figure 15:**
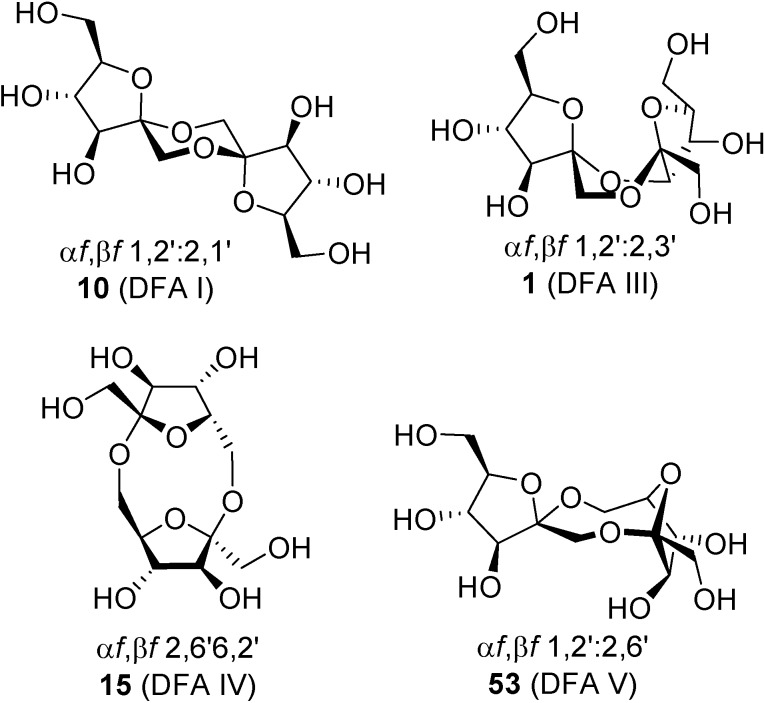
DFA isomers available by biosynthetic routes.

Up to now, four DFA isomers have been obtained by enzymatic degradation of fructans: α-d-fructofuranose-β-d-fructofuranose-1,2’:2,3’-dianhydride (**1**, DFA III), α-d-fructofuranose-β-d-fructofuranose-1,2’:2,6’-dianhydride (**53**, DFA V), α-d-fructofuranose-β-d-fructofuranose-1,2’:2,1’-dianhydride (**10**, DFA I), and the non-spiroketal di-β-fructofuranose-2,6’:6,2’-dianhydride (**15**, DFA IV) (Figure 15). Though enzymatic synthesis is less versatile than chemical, exploitation of biocatalyzed processes for the synthesis of certain isomers may easily overcome synthetic drawbacks, especially concerning scaling up. Most of the reports on enzymatic synthesis of DFAs are related to DFA III and DFA IV. In fact their synthesis has become an issue of industrial interest [98]. Reports on DFA I are more scarce and, so far, the DFA V-forming enzyme has not been characterized.

Isolation of DFA I-forming enzymes from *Arthrobacter* spp. was first reported by Kobayashi and coworkers [105]. The enzyme optimal operating conditions were pH 6 and 40 ºC and it was strongly inhibited by Hg^2+^, Fe^3+^, and other metal cations, a common feature for most of these enzymes [106]. *Streptomyces* spp. have also been shown to be a source of DFA I-forming enzyme [107]. More recently, Haraguchi and coworkers have reported new DFA I-forming enzymes from *Arthrobacter* spp. with slightly prolonged heat stability [108,109], a feature that is mandatory when technical scale synthesis of this isomer is demanded. In the case of DFA V, reports are even more frugal. DFA V has been identified as a transient product during early stages of inulin thermolysis [22] and its structure confirmed as its per-O-acetate [110]. DFA V-producing enzyme has not been isolated yet and only evidences of its actions have been described [111].

Enzymatic preparation is far more developed in the case of DFA III and the non-spiroketalic DFA IV [34,36]. The abundance of reports on isolation, characterization and even gene sequencing and engineering has provided a broader pool of DFAs III and IV-producing enzymes, which might be useful tools to face the ultimate challenge: industrial enzymatic biosynthesis of DFAs. Generally, enzymes dedicated to industrial production require high productivity and thermal stability. Yokota and coworkers have pioneered this field. These authors reported the isolation of an extracellular DFA III-producing IFTase from *Arthrobacter* sp. H65-7 featuring high productivity (up to 90 U mL^-1^) and thermal tolerance (retention of 80% activity after treatment at 70 ºC) [112,113]. Saito and Tomita have also reported the isolation and characterization of a LFTase from *Arthrobacternicotinovarorans* GS-9 well suited for mass production of DFA IV [114]. As a major advantage, enzyme excretion can be stimulated by their specific substrates and are easily purified. Furthermore, substrate conversion yields in a variety of conditions are excellent (Table 6). Minor traces of fructose and short oligofructosides (1-kestose, nystose, and fructofuranosyl nystose) were removed by fermenting the reaction solution with baker yeast. As a drawback, the productive enzyme could not be recycled.

**Table 6:** Enzymatic production of DFA III and IV.

Substrate	Product	Yield (%)
inulin, 50 g/L	DFA III	93.0
inulin, 250 g/L	DFA III	82.5
levan, 20 g/L	DFA IV	75.5
levan, 40 g/L	DFA IV	73.3

Recently, enzymes featuring improved thermal stability have been reported. In the particular case of DFA III-forming enzymes, although temperature range has not been significantly raised, enzymes remain active after prolonged heating [115,116]. In all cases, substrate selectivity and product outcome profile still remain exquisite.

Similarly, improvements on LFTases performance in DFA IV formation have been achieved. However, only in few of these cases are the DFA yield and purity as high as in the case of the inulin converting enzymes [117]. These enzymes are generally more easily inactivated by the presence of metal cations (Mn^2+^, Cu^2+^, Fe^3+^ or Ag^+^) [118] and are very sensitive to substrate origin (molecular weight and branching) [119,120].

The search for efficient DFA-producing enzymes has lead to the development of a DFA IV production system directly from sucrose in a single culture by using a levan producing *B. subtilis* as a host strain for the expression of LFTase gene [121]. Furthermore, intensive efforts have led to the isolation of new enzymes, such as those reverting DFA formation [122].

As evidence of the increasing interest in the development of DFA producing technologies, in the last decade, a number of genes encoding for DFA-forming enzymes (IFTases and LFTases) have been cloned [123,124]. Gene homology evaluation and site-directed manipulation might open new possibilities to engineer enzymes producing additional DFA isomers or featuring improved performance. A genetically engineered *E. coli* has been reported to produce a DFA III-forming enzyme with twice as much activity compared to that of the original strain [125]. A similar modification in LFTase-encoding gene resulted in a 5-fold increase on the DFA IV-forming enzyme activity [126]. However, although these evident advances increased enzyme production, they did not improve enzyme turnover.

Lee and coworkers have tried to improve enzyme turnover by immobilizing a LFTase on different solid supports [127]. Immobilized enzyme activity was comparable to that of the enzyme in solution and, in the best conditions, 60% of the activity was preserved after 20 catalytic cycles. Haraguchi and coworkers have also reported a heat-stable immobilized IFTase, which activity is virtually unaffected after repeated use (up to 8 cycles) [128]. The authors suggest that a reactor using this immobilized enzyme might be envisioned for industrial production of DFA III. More recently, aiming at developing a method for commercial production of DFA III, Vorlop and coworkers have reported a genetically engineered enzyme tolerant to prolonged heating and, thus, well suited to function in continuous processes [129]. Enzyme expression in *E. coli* was extremely efficient (1760 U mL^-1^). Moreover, the enzyme could be entrapped in calcium alginate hydrogels to enable the preparation of homogeneous enzyme-active beads (196 U mL^-1^).

## Conclusions and Perspectives

Difructose dianhydrides (DFAs), a unique class of spiroketal disaccharides, have remained laboratory curiosities until their presence in significant proportion in food materials, such as caramels or chicory, was reported. This discovery represented a milestone in DFA chemistry, fuelling intense research that has revealed the biological, nutritional, or technological relevance of this family of compounds. The development of efficient methodologies to access the individual members is crucial to unravel their biological role and develop new applications. Pursuing this aim, chemical and enzymatic syntheses have long followed disparate paths. However, as revealed in this account, enzymatic and chemical synthetic strategies are complementary. Considering the broad spectrum of spirocyclic structures arising from cyclodimerazition of fructose, it is impressive that the difructose dianhydride isomers that can be selectively synthesized via either one of these routes account for more that 90% of the disaccharidic fraction of fructose caramel. A variety of strategies have been implemented to obtain DFA derivatives aiming at new or improved properties. In the era of functional food products and with such tools in hand, further interesting perspectives might be envisioned for difructose dianhydrides, provided that a complete mapping of their biological and nutritional features is completed. The extraordinary body of knowledge on DFAs accumulated within the last decade from diverse fields can now be put forward to obtain new isomers, discover new enzymes, determine new biological functions and develop new applications or production methodologies.
